# Toward a standard preoperative MRI protocol for functional neurosurgery

**DOI:** 10.1162/IMAG.a.52

**Published:** 2025-06-24

**Authors:** Christopher Güttler, Johannes Achtzehn, Patric Blomstedt, Stéphan Charbardès, Katharina Faust, Stefan Hetzer, Brian H. Kopell, Joachim K. Krauss, Andres Lozano, Joseph Neimat, Francisco A. Ponce, Pratik Rohatgi, John Rolston, Mathieu Santin, Philip A. Starr, Veerle Visser-Vandewalle, Andrea A Kühn, Andreas Horn, Anna Tietze

**Affiliations:** Institute of Radiology, Charité - Universitätsmedizin Berlin, Corporate Member of Freie Universität Berlin, Humboldt-Universität zu Berlin, and Berlin Institute of Health (BIH), Berlin, Germany; Movement Disorders and Neuromodulation Unit, Department of Neurology, Charité - Universitätsmedizin Berlin, Corporate Member of Freie Universität Berlin, Humboldt-Universität zu Berlin, and Berlin Institute of Health (BIH), Berlin, Germany; Dept of Clinical Neuroscience / Unit for Deep Brain Stimulation, University Hospital of Northern Sweden, Umeå, Sweden; Department of Neurosurgery, Université Joseph Fourier, Grenoble, France; Department of Neurosurgery, Charité - Universitätsmedizin Berlin, Corporate Member of Freie Universität Berlin, Humboldt-Universität zu Berlin, Berlin Institute of Health, Berlin, Germany; Berlin Center for Advanced Neuroimaging, Charité – Universitätsmedizin Berlin, Berlin, Germany; Nash Family Center for Advanced Circuit Therapeutics, Icahn School of Medicine at Mount Sinai, New York, NY, United States; Department of Neurosurgery, Hannover Medical School, Hannover, Germany; Division of Neurosurgery, Department of Surgery, University of Toronto, Toronto, Ontario, Canada; Department of Neurosurgery, University of Louisville, School of Medicine, Louisville, KY, United States; Department of Neurosurgery, Barrow Neurological Institute, St. Joseph’s Hospital and Medical Center, Phoenix, Arizona; Department of Neurosurgery, Boston University, Boston, MA, United States; Department of Neurosurgery, University of Utah, Salt Lake City, UT, United States; Sorbonne Université, Institut du Cerveau - Paris Brain Institute - ICM, INSERM, CNRS, Paris, France; ICM, Centre de NeuroImagerie de Recherche - CENIR, Paris, France; Weill Institute for Neurosciences, University of California, San Francsico, CA, United States; Department of Neurosurgery, University of California, San Francisco, San Francisco, CA, United States; Network Stimulation Institute, Department of Stereotactic and Functional Neurosurgery, University Hospital Cologne, Germany; NeuroCure Cluster of Excellence, Charité - Universitätsmedizin Berlin, Berlin, Germany; Center for Brain Circuit Therapeutics Department of Neurology Brigham & Women’s Hospital, Harvard Medical School, Boston, MA, United States; MGH Neurosurgery & Center for Neurotechnology and Neurorecovery (CNTR) at MGH Neurology Massachusetts General Hospital, Harvard Medical School, Boston, MA, United States; Institute of Neuroradiology, Charité-Universitätsmedizin Berlin, Corporate Member of Freie Universität Berlin, Humboldt-Universität zu Berlin, and Berlin Institute of Health (BIH), Berlin, Germany

**Keywords:** deep brain stimulation, Parkinson’s disease, dystonia, essential tremor, magnetic resonance imaging, protocol harmonization, targeting

## Abstract

Deep Brain Stimulation (DBS) is a well-established approach to treat movement disorders such as Parkinson’s Disease, dystonia or essential tremor. For optimal therapy response, accurate electrode placement is critical requiring high signal-to-noise of target areas in preoperative MRI. Currently, imaging protocols vary considerably between DBS centers, making it difficult to compare results or pool data for research purposes. Here, various currently employed MRI sequences from several DBS centers are evaluated regarding their suitability for DBS targeting and a protocol is suggested taking image quality and practical considerations into account. Two healthy subjects (52-year-old female and a 37-year-old male) were each scanned with various sequences (5 T2w, 1 PDw, 4 T2FLAIRw, 2 T2*w, 5 SWI, 2 FGATIR, 1 T1TIR, and 2 QSM techniques) that then were rated by 12 experienced DBS surgeons for their suitability for targeting the subthalamic nucleus (STN), the internal globus pallidus internus (GPi), and the ventrointermediate (VIM) thalamic nucleus. For a subset of sequences, surgeons were asked to identify the optimal DBS target in the STN and GPi. Contrast-to-noise ratios (CNR) were calculated and correlated to intra-rater z-scores and distances of target coordinates. For STN-DBS, surgeons rated T2w, most SWI, QSM, and T2FLAIRw the highest. For GPi-DBS, FGATIR, PDw, and SWI and for VIM-DBS, FGATIR were deemed the most suitable. Higher CNR correlated with higher intra-rater z-scores (R^2^= 0.29, p < .005) which improved targeting (R^2^= 0.18, p < .05). Our MRI protocol suggestion is a first step toward standardizing preoperative imaging. All imaging data, MRI sequence parameters, and protocol files are made openly available.

## 1 Introduction

Optimal visualization of Deep Brain Stimulation (DBS) targets is critical for best results in stereotactic surgical planning, which leads to optimal treatment results. In this context, it is key to delineate basal ganglia structures such as the subthalamic nucleus (STN), the internal globus pallidus internus (GPi), as well as intrathalamic nuclei, for example, the ventrointermediate (VIM) thalamic nucleus, with the highest possible contrast. These three nuclei represent the most common DBS targets and have been used to treat brain disorders such as Parkinson’s Disease, dystonia, essential tremor, Obsessive Compulsive Disorder (OCD), and Tourette’s Syndrome (Deuschl et al., 2006;Kremer et al., 2021;Mallet et al., 2008).

To optimally demarcate these deep lying grey matter structures, strong contrast of their anatomical borders, that is, strong CNR is critical. Multiple concepts have been proposed to increase contrast in these structures (basal ganglia and thalamus). One concept implies nulling the adjacent white matter signal (Tourdias et al., 2014), and today many DBS centers use the Fast Gray Matter Acquisition T1 Inversion Recovery (FGATIR) (Sudhyadhom et al., 2009), white matter attenuated inversion recovery (WAIR) (Vassal et al., 2012), or WM nulled sequences (Tourdias et al., 2014), which shows similar contrast properties. A second concept involves detecting iron depositions to enhance contrasts. This strategy is included in susceptibility-weighted imaging (SWI) or the quantitative susceptibility mapping approach (QSM) (Rasouli et al., 2018).

As mentioned, optimal depiction of target structures is important for stereotactic planning. After surgery, the same applies to postoperative electrode localizations (Horn et al., 2019), which has become increasingly applied to guide DBS programming (Roediger et al., 2022) and statistical analyses on a group level (Treu et al., 2020) that may involve localized sweet spot mappings (Dembek et al., 2019) and connectomic analyses (Horn & Fox, 2020). DBS electrodes themselves are often reconstructed on computed tomography (CT), which offers a combination of optimal contrast (electrode vs. brain tissue), sub-millimeter resolution, and minimal distortion artifacts. However, while using CT one can reconstruct the electrodes excellently, to draw conclusions about their exact location relative to surrounding anatomy, high-quality preoperative MRI is again crucial (Krauss et al., 2021;Treu et al., 2020). To facilitate this process of electrode localizations, specialized imaging software has been created, which fuses pre- and postoperative imaging data (Neudorfer et al., 2023;Noecker et al., 2021).

Obviously, in both pre- and postsurgical settings, the reliability of results hinges upon accurate visualization of targets. Most DBS centers have come up with a locally curated preoperative imaging protocol in collaboration with their radiology and MR-physics departments or with other DBS centers (Gravbrot et al., 2020;Hariz et al., 2004;Tourdias et al., 2014). Across institutions, however, choices of preoperative sequences substantially vary. Indeed, it is reasonable to assume that there are rarely two DBS centers across the globe that acquire the exact same preoperative imaging protocol. To the best of our knowledge, no standardized protocol has been proposed—and while colleagues are usually very open to informally sharing their protocols across centers, to our knowledge, no protocol has been made publicly available (i.e., deposited in a data repository) so far. In other imaging domains, MR protocols have been openly shared, such as the advanced diffusion and functional MRI multiband protocols (Van Essen et al., 2012) developed by the Human Connectome Project (https://www.humanconnectome.org/hcp-protocols). To our knowledge, this open practice has resulted in many imaging studies adopting a standardized protocol. This may lead to harmonization of data across centers, which could be critical to analyze large amounts of data pooled across institutions (Biswal et al., 2010).

Also, in the field of DBS, data sets and clinical outcomes are now frequently pooled across centers, especially for rare DBS indications. One prominent example is the DBS for Tourette’s Disease Registry dataset (https://tourettedeepbrainstimulationregistry.ese.ufhealth.org/), which has been successfully set up to give investigators access to a larger sample of patients with the aim to improve therapy (Deeb et al., 2016). Since each center usually only operates on a handful of patients in this disorder, it is impossible to correlate stimulation sites with clinical effects using monocentric data. Instead, the registry initiative has made this possible, resulting in insightful publications that will refine treatment in the next generation of patients worldwide (Johnson et al., 2020,^2021^). Similar examples of pooled analysis exist in other diseases, such as dystonia (Horn et al., 2022;Reich et al., 2019), essential tremor (Nowacki et al., 2022), OCD (Li et al., 2020,^2021^), cluster headache (Nowacki et al., 2020), or Alzheimer’s Disease (Germann et al., 2021;Ríos et al., 2022). Most of these examples have been analyzed with the same processing pipeline (Neudorfer et al., 2023;Treu et al., 2020), that is, a certain standard of data analysis has emerged. However, a key limitation of all mentioned studies is the lack of harmonization in the underlying preoperative MRI data.

This issue has been noticed by the community and has, for instance, emerged in multiple informal discussions between experts present at the DBS Think Tank conference, which is annually organized by the center in Gainesville at University of Florida (Vedam-Mai et al., 2021). A key challenge of harmonizing preoperative imaging is that different surgeons follow different concepts for deriving the target, for example, different landmarks are used for indirect targeting or different MRI sequences employed for direct targeting. New insights and developments make this an ongoing process and continuous exchange across centers is necessary for sequence and protocol optimization. In other words, it will unlikely be feasible to create a single protocol that suits the needs of every single DBS center across the globe.

Given these thoughts, we still decided to take a very first step along the journey and propose a first draft for a preoperative DBS imaging protocol. Our results do not represent a final and definite version. Instead, we believe this could become a dynamic and continuously developing process to which we now provide a starting point. To obtain this first suggestion, we scanned two subjects of different age and gender with a large battery of sequence suggested by three DBS and two Neuroimaging Research Centers (total scan time approx. 3.5 hrs). We then loaded the imaging data into an online visualization and scoring tool that was deliberately created for this study. Using this tool, each of the 22 sequences were rated by 12 neurosurgeons from 11 DBS centers regarding suitability to target each of the three most common structures (STN, GPi, and VIM). Finally, we asked neurosurgeons to localize DBS targets on various sequences with the aim to quantify their clinical utility. This evaluation, conducted by the neurosurgeons, took place in a single sitting for each participant. The objective was to acquire comprehensive and high-quality datasets while simultaneously ensuring consistency in the assessment process.

Based on these ratings, we were able to condense the lengthy protocol into three that could be applied in clinical practice (total scan time for the STN 36 min, for the GPi and VIM 20 min). This protocol suggestion along with all imaging data are made openly available in a data repository with the aim to get discussions and further suggestions going. Once published, we hope to receive feedback from centers world-wide, to revise the protocol, and to be able to repeat the same procedure for further refinements of the protocol.

## 2 Methods

### 2.1 Imaging sequence selection

Standard MRI protocols for DBS targeting were collected from three DBS centers (Charité – University Medicine Berlin, and Würzburg University Hospital, Germany, Aarhus University Hospital, Denmark) and two Neuroimaging Research Centers (Paris Brain Institute, Hôpital Pitié-Salpêtrière, France, and Berlin Center for Advanced Neuroimaging – Charité University Medicine Berlin, Germany). In cases of overlapping sequences, that is, with very similar imaging parameters, only one was selected leading to a final imaging protocol consisting of five T2w, one PDw, four T2FLAIRw, two T2*w, five SWI, two FGATIR, one T1TIR, and two QSM techniques, one based on multi-parameter mapping (Cooper et al., 2020), the other using a QSM approach (Santin et al., 2017).

### 2.2 Subjects and imaging protocol

The study was approved by the local ethics committee of Charité – Universitätsmedizin Berlin (master vote #EA1_102_21). Two healthy subjects, notably without known neurological or psychiatric disorders, were included after giving written consent: a 52-year-old female and a 37-year-old male. Scanning took place on three separate occasions and was carried out on Skyra 3T Siemens system using a 64 Channel receive-only headcoil (Siemens, Erlangen, Germany). All sequences were acquired for both subjects, except for sequences 3, 8, 9, 14, 15, 20, and 22. These sequences were either added later in the study or are no longer in use and were therefore evaluated only once. An overview about selected sequence parameters is given inTable 1. Detailed sequence protocols can be downloaded from osf.io (DOI: 10.17605/OSF.IO/P63ZF) in PDF format along with EXAR files that can be uploaded directly into Siemens MRI systems (software version XA30).

**Table 1.** Overview of scan sequence parameters.

Technique	ID	Sequence	TR/TE (ms)	Inversion time (ms)	Flip angle ( ^∘^ )	Resolution (mm)	FOV	Slice direction	Acceleration	Time (min., sec.)
T2	01	T2 TSE TRA 2 mm	13320/101	NA	150	0.7*0.7*2.0	250	Axial, 2D	n/a	4’15’’
	02	T2 TSE TRA 2 mm	3600/81	NA	120	0.7*0.7*2.0	220	Axial, 2D	n/a	4’28’’
	03	T2 SPACE SAG P2 ISO	2500/232	NA	var	1.0*1.0*1.0	256	Sagittal, 3D	GRAPPA 2	8’02’’
	04	T2 TSE AX 384 1.5 mm	4430/80	NA	150	0.6*0.6*1.5	240	Axial, 2D	GRAPPA 2	14’56’’
	05	T2 TSE 1.5 mm	5590/80	NA	150	0.6*0.6*1.5	240	axial, 2D	GRAPPA 2	20’42’’
T2FLAIR	06	T2 FLAIR	7200/81	2250	150	0.7*0.7*2.0	220	Axial, 2D	GRAPPA 2	5’33’’
	07	T2 BLADE TRA dark fluid	8000/146	2370	180	0.8*0.8*5.0	240	Axial, 2D	GRAPPA 2	4’02’’
	08	T2 SPACE DA-FL SAG P2 ISO	5000/388	1800	120	1.0*1.0*1.0	256	Sagittal, 3D	n/a	11’40”
	09	T2 SPACE DA-FL SAG P2 ISO	5000/388	1800	120	1.0*1.0*1.0	256	Sagittal, 3D	n/a	12’48’’
PD	10	PD TSE TRA 2 mm	3600/11	NA	120	1.0*1.0*2.0	250	Axial, 2D	GRAPPA 2	3’34’’
T2*	11	T2 FL2D TRA HEMO	625/25	NA	30	0.4*0.4*2.0	192	Axial, 2D	GRAPPA 2	6’20’’
	12	T2 FL2D TRA HEMO	625/25	NA	30	0.5*0.5*2.0	192	Axial, 2D	GRAPPA 2	4’17’’
SWI	13	T2 SWI3D TRA P2	27/20	NA	15	0.8*0.8*2.0	200	Axial, 2D	GRAPPA 2	4’32’’
	14	T2 SWI3D TRA P2 1.15 mm 384	27/20	NA	15	1.1*1.1*1.2	220	Axial, 2D	GRAPPA 2	5’31’’
	15	T2 SWI3D TRA P2 FOV PHASE 100	27/20	NA	15	0.8*0.8*2.0	220	Axial, 2D	GRAPPA 2	4’32’’
	16	T2 SWI3D TRA P2 1.2 mm 384	28/20	NA	15	0.6*0.6*1.2	220	Axial, 2D	GRAPPA 2	6’37’’
	17	T2 SWI3D TRA P2 1.2 mm 384	28/20	NA	15	0.6*0.6*1.2	220	Axial, 2D	GRAPPA 2	7’10’’
T1 TIR	18	T1 TIR TRA	2500/13	499	150	1.0*1.0*2.0	256	Axial, 2D	n/a	4’27’’
FGATIR	19	FGATIR_TR3000	3000/3.44	414	8	0.9*0.9*1.0	240	Axial, 3D	n/a	9’08’’
	20	FGATIR	3000/3.41	414	8	1.0*1.0*1.0	256	Axial, 3D	n/a	11’14’’
Mapping	21	SMPM FL3D SAG 1 mm	23/2.46; 4.92; 7.38; 9.84; 12.30; 14.76; 17.22; 19.68; 23.0	NA	6	1.0*1.0*1.0	256	Sagittal, 3D	GRAPPA 2	6’44’’
	22	ICM_GRE	39/3; 6; 9; 12; 15; 18; 21; 24; 27; 30; 33; 36;	NA	15	1.0*1.0*1.0	224	Sagittal, 3D	GRAPPA 2	8’21’’

Further details, including additional parameters required to distinguish certain sequences, can be found in the sequence parameter sheets (DOI: 10.17605/OSF.IO/P63ZF).

TR: repetition time; TE: echo time; FOV: field of view.

### 2.3 Construction and application of an online rating tool

All acquisitions were pseudonymized and converted to NIfTI (Neuroimaging Informatics Technology Initiative) format. The optimal viewing contrast as well as the most representative axial slice numbers for STN, or, in case of the FGATIR sequences, GPi targeting were determined for each sequence by an experienced board-certified neuroradiologist with > 10 years of clinical experience (AT) and saved for later use.

**Fig. 1.**
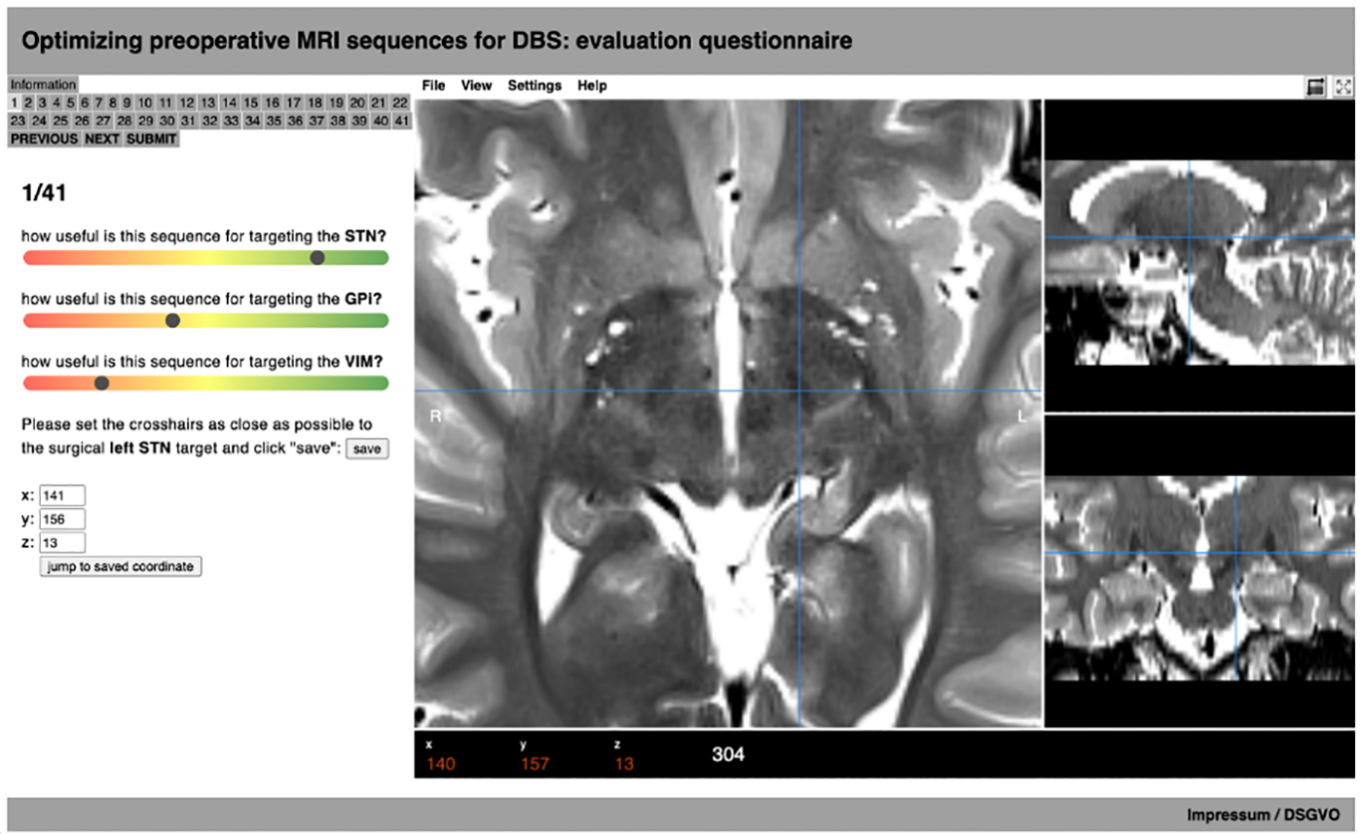
Screenshot of the rating tool. Participants were asked to rate the usefulness of each specific acquisition for targeting the the subthalamic nucleus (STN), globus pallidus internus (GPi), and ventrointermediate (VIM) thalamic nucleus on visual analogue scales as well as to mark a single surgical target (STN vs. GPi, depending on the sequence).

A website (Fig.1, HTML/CSS/JavaScript/PHP) with built-in medical image viewing capabilities using the Papaya viewer (https://github.com/rii-mango/Papaya) was created. The landing page provided information about how to navigate the website and use the built-in image viewer. It also allowed users to enter their contact information and general comments.

All acquisitions (mixed across the two subjects) were presented to each user in randomized order using the previously identified suggested windowing and location as a starting point. Participants then scrolled through the 3D volumes and had the opportunity to adjust the windowing according to their own preferences with the goal in mind to optimally visualize DBS targets in the STN, GPi, and VIM regions, respectively. Next, participants were asked to rate each imaging sequence on a visual analogue scale according to “how useful is this sequence for targeting the STN/GPi/VIM?”. The rating scale (Fig. 1) was color-coded from red to green and internally converted to a numerical value ranging from 0 to 100, with 0 (green) being on the far left and 100 (red) on the far right of the scale.

Finally, for each sequence, participants placed the viewers’ crosshair onto one surgical target on the left hemisphere (STN vs. GPi). A preselection to choose the target for each sequence was carried out by the core authors, for example, on T2-weighted sequences, we asked surgeons to target the STN, while on FGATIR-sequences, we asked them to target the GPi. Twenty-nine DBS surgeons from 24 centers were asked to participate in the rating process, of which 12 completed the questionnaire.

### 2.4 Data processing and statistical analysis

All data were processed using custom-written Matlab code (Version 9.11, The MathWorks Inc.). Statistics were calculated using GraphPad Prism 9.4.0.

#### 2.4.1 Qualitative image assessment by raters

After de-randomization, all user rating input data were intra-rater z-transformed. The summary distribution of rating results is presented as violin plots for each sequence and for each target, that is, STN, GPi, VIM, separately. The y-axis represents the intra-rater z-scores for each sequence, with the median of each violin plot corresponding to the median normalized score for that sequence across participants. T-tests were performed to compare ratings of different sequences regarding STN targeting head-to-head. P-values were adjusted for multiple comparisons using the Tukey method, and the family-wise alpha threshold was set to 0.05.

#### 2.4.2 Assessment of image quality metrics

Quantitative CNR were calculated for each sequence using MRIQC (Esteban et al., 2017), a software toolbox which consists of an automated nypipe workflow (Gorgolewski et al., 2011) that includes skull stripping, spatial normalization, head mask calculation, and brain tissue segmentation and generates reports with statistical summaries and visualizations. Simple linear regression was used to examine the relationship between CNR and intra-rater z-score, CNR and distance between each coordinate and coordinates averaged across raters (‘distance’, mm). Coefficients of determination (R^2^) and p-values were reported.

## 3 Results

### 3.1 Qualitative image assessment by raters

**Fig. 2.**
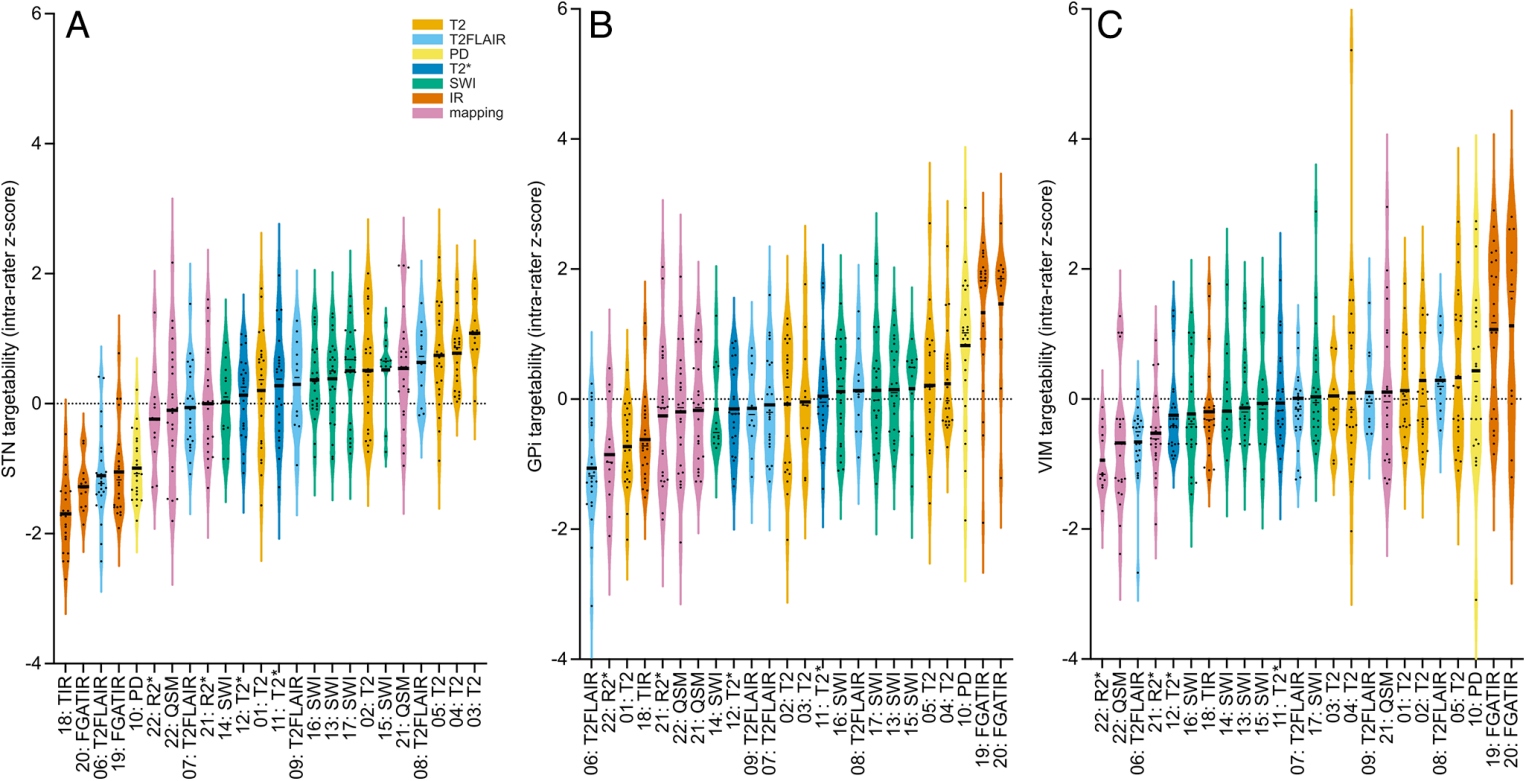
Usefulness of MRI sequences for targeting (A) the subthalamic nucleus (STN), (B) the globus pallidus internus (GPi), and (C) the ventrointermediate (VIM) thalamic nucleus. All data were intra-rater z-transformed, and additional thick lines mark the group average of the ratings. The different sequences and their numbers on the x-axes correspond to the numbering inTable 1; similar sequence types are grouped by color.

Images can be accessed in NIfTI format at osf.io (DOI: 10.17605/OSF.IO/P63ZF).Figure 2summarizes the rating results for the STN (Fig. 2A), GPi (Fig. 2B), and VIM (Fig. 2C). Details are specified inTables S1 and S2. All T2w, most SWI sequences, including the MPM-based QSM, and the 3D T2FLAIRw sequence with shorter TE were rated the most suitable for STN targeting. For the GPi, both FGATIR sequences stood out and were followed by the PDw sequence. The SWI with almost isometric voxels (1.1*1.1*1.2 mm^3^) appeared to be useful, too, opposed to the non-isometric SWI versions that were rated much lower. For the VIM, results were even clearer; the FGATIR sequences were preferred by all raters, whereas all other sequences were rated average or not suitable. Correlation and two-tailed paired t-test analyses of the ratings across all sequences scanned in both subjects showed a highly significant correlation between subjects (STN: R = 0.89, p < .001; GPi: R = 0.72, p < .0005, VIM: R = 0.89, p < .0001). No significant differences were observed between subjects (STN: p > .054; GPi: p > .47; VIM: p > .60), supporting the data’s representativeness even with the small sample size (Fig. S1).

**Fig. 3.**
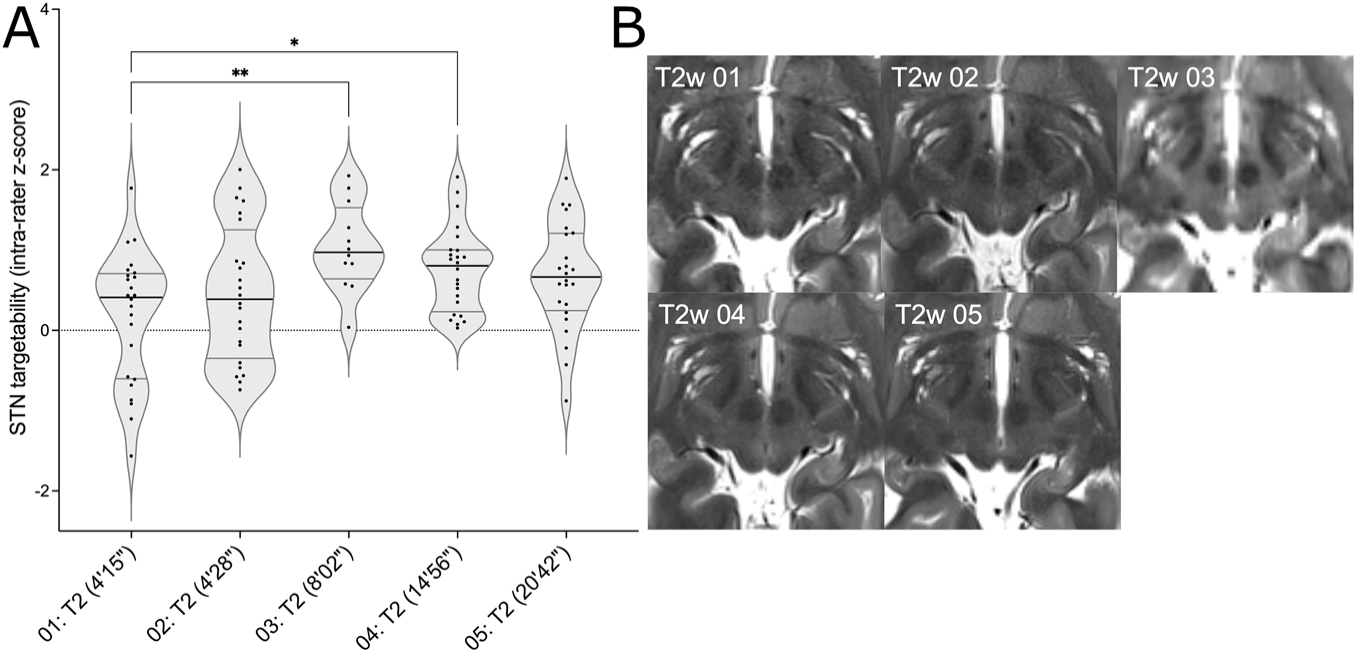
(A) Ratings (intra-rater z-scores) of T2w sequences for the usefulness of subthalamic nucleus (STN) targeting. Asterisks indicate level of statistical significance: * p ≤ .05, ** p ≤ .01. Acquisition times are specified for each version and it is evident that longer acquisitions do not automatically translate into higher ratings. (B) Representative slices of T2 TSE version 01 (4’15’’), T2 TSE version 03 (4’28’’), T2 SPACE version 03 (8’02’’), T2 TSE version 04 (14’56’’), and T2 TSE version 05 (20’42’’).

Since ratings were less clear for the STN visibility than for the GPi and VIM, a detailed comparison between the different T2w versions was amended.Figure 3Ashows ratings for the 5 versions (illustrated inFig. 3B), including acquisition times which ranged from 4:15 to 20:42 min. Compared to the fastest T2w version, versions 03 and 04 were rated significantly better (p < .01 and p < .05 respectively). Improved STN visibility must, however, be balanced with longer acquisition times, in the case of version 03 with 1.9 and version 04 with 3.5 times longer scanning times (8:02 min vs. 14:56 min) compared to the 4:15 min of version 01. This is particularly true when scanning movement disorders patients, where shorter acquisition times are particularly suitable. Of note, the very long acquisition time of version 05 (20:42 min) did not translate into significantly better ratings (p > .05). Although version 01 was rated the worst among T2w sequences, it was still relatively well suited for STN targeting and superior to, for example, SWI, version 16, and T2 SPACE DA FL, version 09 (corresponding to a 3D T2FLAIR sequence), lasting 6:37 min and 12:48 min, respectively.

### 3.2 Assessment of image quality metrics

**Fig. 4.**
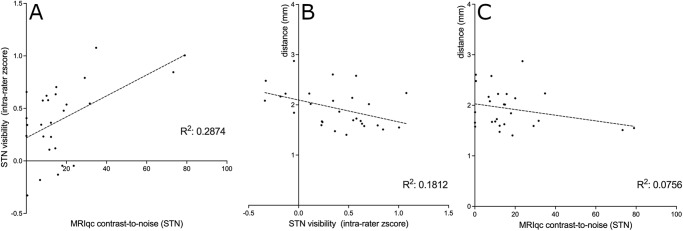
(A) A better CNR results in higher intra-rater z-scores, that is, in better sub thalamic nucleus (STN) visibility. (B) Higher subjective ratings with higher intra-rater z-scores correlate with lower variance in surgical targeting, which is expressed as the distance to the respective center of all coordinates across raters (in mm). (C) The variance in surgical targeting correlates weakly with the CNR.

Higher CNR correlated significantly with intra-rater z-scores (R^2^= 0.29, p < .0039), that is, a better CNR ratio resulted in better ratings regarding STN visibility (Fig. 4A). This applied also for image resolution and intra-rater z-scores (in-plane resolution: R^2^= 0.12, p < .0295; slice thickness: R^2^= 0.14, p < .0158). The improved STN visibility translated into more accurate and precise STN targeting, expressed in terms of smaller variance (distance to the mean coordinate in mm) across raters (R^2^= 0.18, p < .0239;Fig. 4B). The two datapoints with the highest MRIQC CNR and a high visibility rating are the sequences 4 and 5. Both are 1.5 mm T2-TSE sequences with a very long scan duration of 14’56’’ and 20’42’’ and a very high contrast. The removal of these two datapoints would result in a decrease of R^2^from 0.29 to 0.14 and an increase of the p-value from < .0039 to a p-value of > .067. It should, however, be noted that this high CNR requires accepting a clinically impractically long acquisition time. The correlation between STN targeting and the CNR was weak (Fig. 4C; R^2^= 0.08, p > .1651), as was the correlation between STN targeting and image resolution (in-plane resolution: R^2^= 0.08, p > .1375; slice thickness: R^2^= 0.02, p > .4437). This suggests that additional image characteristics, beyond what can be automatically calculated, are relevant for targeting the STN. No significant correlation was found between the CNR and visibility ratings for either GPi (p < .63, R^2^= 0.006) or VIM (p < .06, R^2^= 0.09).

## 4 Discussion

The following conclusions can be drawn from the present study. First, there was a significant difference between sequences to identify DBS targets and a few sequences clearly stood out. Further, longer acquisition times did not automatically result in better image quality and usefulness for targeting. Second, automated measures of CNR (Esteban et al., 2017) correlated with expert surgeon ratings. However, only the ratings (and not quantitative CNR measurements) correlated significantly with the reliability of how surgeons targeted the STN, implying that expert opinions were more apt in assessing MRI sequence quality than automatically calculated quality measures. Still, automated CNR measures could play a role in preselecting or further optimizing sequences, although we would not recommend using MRIQC for assessing sequences for targeting GPi or VIM, since the GM/WM CNR metric showed no significant correlation with visibility ratings for these structures (while it did correlate for the STN target). Our results allow the configuration of dedicated DBS protocols that we propose as a first version (Table 2andFig. 5). Here, we aim at a compromise between best possible visibility of target structures and scan durations. Notably, some sequences may yield better results, which must, however, be balanced against longer acquisition times associated with a higher risk of movement artefacts or longer anaesthesia times and, finally, the considerate use of health care resources. We chose to not include the 20-min. T2w sequence in the protocol, as it offered no significant advantages over a 15-min. or even 8-min. sequence. This choice may help reduce overall scan time and facilitate clinical implementation of this protocol for institutions implementing our suggestions. Sequence 3, a 3D T2w SPACE was acquired in only one of the two subjects because it was added later in our experiment. Nevertheless, it proved highly valuable for STN identification, receiving high ratings from most participants, and was therefore included in our final protocol suggestion. Our findings indicate that SWI is particularly useful for identifying the STN, whereas the FGATIR sequence was clearly superior for visualizing the GPi and VIM compared to all other sequences. For this reason, we excluded the SWI for the GPi/VIM visualization inFigure 5. Although the QSM sequence received high ratings from some participants, there was significant variability in its ratings. Additionally, mapping techniques using phase data (QSM) or magnitude data from multi-echo GRE sequences (R2*) currently require off-line post-processing, which is not readily available at all sites. Furthermore, these methods may be less familiar to some neurosurgeons, which is why we did not include them in our current protocol suggestions.

**Table 2.** Suggested protocols to target the subthalamic nucleus (STN), the internal globus pallidus (GPi), and the ventrointermediate nucleus (VIM).

Target structure	Suggested sequences	Resolution (mm)	Acquisition time
STN	T2w (03)	1.0*1.0*1.0	8’02’’
	T2FLAIR (08)	1.0*1.0*1.0	11’40’’
	SWI (15)	0.8*0.8*2.0	5’31’’
	MPRAGE without/with contrast	0.9*0.9*0.9	2*5’21’’
Gpi	FGATIR (19)	0.9*0.9*1.0	9’08’’
	PD (10)	1.0*1.0*2.0	3’34’’
	MPRAGE without/with contrast	0.9*0.9*0.9	2*5’21’’
VIM	FGATIR (19)	0.9*0.9*1.0	9’08’’
	MPRAGE without/ with contrast	0.9*0.9*0.9	2*5’21’’

MPRAGE, a 3D T1w sequence, without and with gadolinium contrast is added for navigation purposes.

**Fig. 5.**
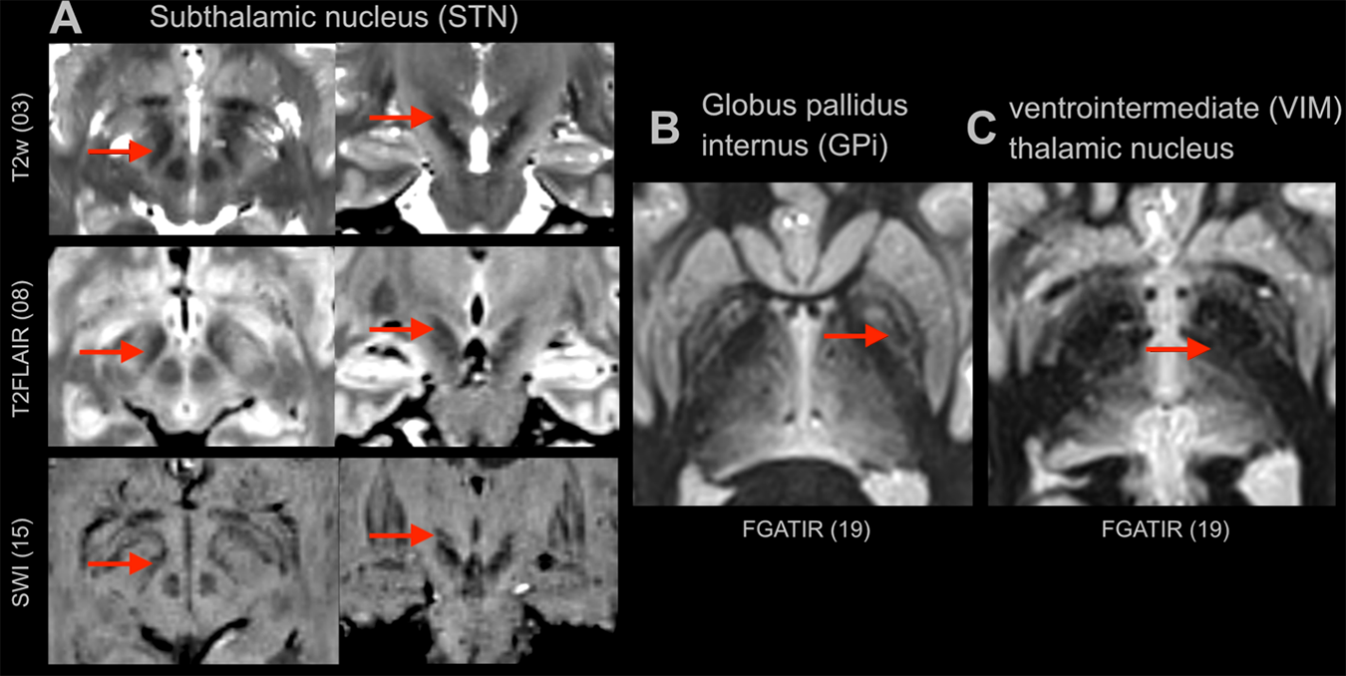
Proposed sequences for the (A) the subthalamic nucleus (STN), (B) globus pallidus internus (GPi), and (C) ventrointermediate (VIM) thalamic nucleus (the MPRAGE for navigation purposes is not shown). For the STN, the three sequences are shown in axial (first column) and coronal (second column) reconstructions; for the GPi and VIM, axial slices are shown. Red arrows indicate the location of the respective target structures.

It is important to consider that the suggested protocols represent only a minimum of MRI sequences and could, of course, be supplemented by sequences that are felt to be necessary by individual surgeons. We make all tested sequences, both longer and shorter versions, openly available as EXAR files, directly importable on Siemens MRI systems, as well as pdf files with detailed scan parameters from osf.io (DOI: 10.17605/OSF.IO/P63ZF). In addition, the MRI data can be downloaded in NIfTI format to get an overview of all included sequences (DOI: 10.17605/OSF.IO/P63ZF).

Our protocol confirmed the truisms that sequences from the family of T2-weighted images are helpful for planning STN-DBS surgery (Boutet et al., 2021;Dormont et al., 2004). A high-resolution T2w is, therefore, essential in PD patients and should be supplemented by SWI, which is sensitive to the magnetic field inhomogeneities caused by iron accumulations (Ward et al., 2014). Experts generally preferred SWI over T2* sequences, although the voxel sizes were similar in our sample. Potentially, the increased susceptibility effects of SWI may render this method superior for STN delineation. In our and others’ experience (Dimov et al., 2018), R2* mapping can further improve STN visibility, but this was not agreed upon by most expert readers, possibly due to the relatively unfamiliar aspect of these images.

Specific importance should be seen in the FGATIR sequences, since—from the ones studied here—these were the only ones capable of visualizing nuclei of the thalamus and visualizing medullary laminae that segregate the internal from the external pallidum. These sequences were highly rated by all experts for VIM and GPi targeting. Out of clinical experience, however, we know that surgeons prefer at least one additional T2w series to delineate thalamus or globus pallidus from the internal capsule, which can obviously be added. FGATIR is a 3D gradient echo sequence with white matter suppression by modifying the inversion pre-pulse and the inversion time to match the WM null-point (Sudhyadhom et al., 2009). This allows the optimal visualization of grey matter structures, especially when they border myelinated structures, such as the GPi that is neighboured by the internal capsule and the medial medullary lamina (and partly divided by the accessory medullary lamina). Of note, in a recent study it was suggested that the FGATIR technique is very well suited to delineate the VIM—a target structure that is otherwise challenging to identify—by suppressing adjacent white matter structures, specifically the ascending dentatorubrothalamic tract (Neudorfer et al., 2022). Finally, comparable sequences, such as the WM-nulled or WAIR sequences, would likely rank similarly to the FGATIR scan based on our experience, since, depending on exact parameters, results between the two sequence families look very similar (Gravbrot et al., 2020;Tourdias et al., 2014;Vassal et al., 2012).

Our study focuses on imaging at 3T, which is, by far, the most commonly applied magnet strength. While 1.5T MRI scanners may have been more available in the past, the signal- and CNR are clearly lower at comparable acquisition times (Cheng et al., 2014). However, data quality might be further maximized by moving to ultra-highfield imaging at 7T (or above). While not adopted by many centers yet, researchers have called the field to do so, based on the notion that many DBS centers are situated in proximity of a 7T magnet close-by (Forstmann et al., 2017). Excitement about acquisitions at 7T may be compromised by the notion that higher field strengths are associated with increased B1 field inhomogeneities and new challenges for distortion correction algorithms (Neumann et al., 2015). Hence, especially when establishing a novel DBS imaging protocol at a 7T, we recommend combining and comparing acquisitions at both 3T and 7T from the same brains to establish protocols. Finally, clinical practicability remains a challenge, as some patients may need to be transferred to research facilities where MRI scans under general anesthesia, which are necessary for certain cases, can be difficult or even impossible.

Several limitations apply to this study. First, the a priori selection of sequences was not carried out in an exhaustive or data-driven fashion. There are many promising work-in-progress sequences (Middlebrooks et al., 2021) with enhanced target structure contrast; however, as they are not yet widely available or require post-processing, we did not include them in our protocol suggestion which is intended to be broadly implementable. Future studies should continue to compare new sequences and techniques using a similar approach. While we asked as many surgeons as possible from different centers to rate results, we did not ask them to contribute their sequences which would have been more objective. As mentioned in the introduction, we hope to revise and curate the suggested protocol further (i.e., to come up with a version 2) after other centers had the chance to test and compare the proposed protocol to their locally favored sequences. For this reason, the protocol should be seen as a first draft and the same study could be repeated easily now that the online rating tool has been created. Similarly, the analysis is based on only two healthy subjects, not on patients that underwent DBS surgery. The scan protocol for each subject was several hours long, and hence was not suitable for patients with PD or other movement disorders who may have had trouble resting still for so long. Another limitation is that MR sequences could not be compared to a CT scan to quantify distortions. Most of the MR sequences tested were derived from DBS protocols that are typically evaluated and aligned with pre- and postoperative CT scans, meaning they have proven reliable in clinical practice. Therefore, we believe that geometrical distortions are unlikely to pose a significant issue in the sequences tested. Nevertheless, this should be formally evaluated once the protocol is implemented clinically. If distortions are quantified, sequences with minimal distortion could be prioritized over those with larger distortions (if present in the protocol). Susceptibility distortions would be related to the water-fat shift of the sequence. Since it is argued that minimizing biases is crucial, a low fat shift (or high spectral width) could potentially guide the choice between two otherwise equally performing methods.

Second, we decided to limit the rating to two healthy subjects and to exclude sequences that were very similar to each other, as we did not want to deter expert readers with a too time-consuming experiment. Our main goal was to attract as many participants as possible and to maintain their motivation and focus during the entire process. We asked them to rate 41 times and believe that we reached a fair compromise between comprehensiveness and workload. Third, we evaluated only the three most common targets and variance in planning coordinates for the VIM was not compared. Other DBS targets such as the centromedian nucleus, cingulate cortex, or the anterior limb of the internal capsule are critical for DBS in other indications and may profit from a similar study focusing on them, specifically. A potential confounding factor is that raters were asked to target only one structure (e.g., the STN) per MRI sequence. This was done to prevent tiring the raters, as asking raters to target 3 structures across 40+ sequences would have required too many ratings in a reasonable time and/or a single sitting. While this could have biased raters toward specific sequences (e.g., rating T2w sequences higher for the STN), it is important to note that some structures, like the STN, are not well visualized on FGATIR or T1w. While we see the most value in intra-sequence class comparisons (e.g., identifying the best T2 scan), inter-sequence class comparisons still offer useful insights. Finally, our study was not geared toward answering the question whether selecting optimal sequences for consistent targeting would lead to improved clinical outcomes in DBS surgeries. However, existing literature consistently shows that accurate electrode placements correlate with better clinical outcomes (Akram et al., 2017;Bot et al., 2018;Horn, 2019;Horn et al., 2019,^2022^;Neudorfer et al., 2023;Nowacki et al., 2022). For instance,Middlebrooks et al. (2024)demonstrated that surgical outcomes for patients with essential tremor were superior when DBS surgeries were planned with 7T scans versus 3T scans, conducted by the same team at the same center.

In conclusion, with the aim to harmonize MRI protocols between DBS centers we suggest a first version that can be easily implemented by importing the provided sequences on individual MRI scanners from osf.io (DOI: 10.17605/OSF.IO/P63ZF). This proposal is based on the rating of 12 international DBS experts as well as practical considerations such as acquisition time and widespread availability of sequences. While STN targeting is currently best accomplished by using T2w/T2-FLAIR sequences as well as susceptibility-weighted techniques, GPi and VIM are optimally delineated on FGATIR. For practical purposes and if resources at the MRI scanner facility allow for that, a general DBS protocol could be implemented, including T2w, T2FLAIR, SWI, and FGATIR, supplemented by 3D T1w navigation sequence.

The key next steps of this endeavour are as follows: First, we hope that DBS centers will employ our suggested protocol and compare it with their current ones. If certain sequences from their local protocols perform better, we welcome the opportunity to collect the optimized sequences for a second round of evaluations. This concept will be communicated via our network(s) once the study is published. In parallel, adapting our protocol for other MRI vendors will be important, and we hope to receive support from the community in this effort, specifically by manually adapting sequences on other MRI systems and sharing the protocols in the central repository (10.17605/OSF.IO/P63ZF).

With this work, we hope to facilitate the harmonization of MRI protocols and, in consequence, data pooling to promote research possibilities as well as improved clinical outcomes in the field of DBS.

## Supplementary Material

Supplementary Material

## Data Availability

We make all tested sequences, both longer and shorter versions, openly available as an EXAR files, directly importable on Siemens MRI systems, as well as pdf files with detailed scan parameters from osf.io (DOI: 10.17605/OSF.IO/P63ZF). In addition, the MRI data can be downloaded in NIfTI format to get an overview of all included sequences (DOI: 10.17605/OSF.IO/P63ZF).
