# Supplementary material for: Toward a standard preoperative MRI protocol for functional neurosurgery
Source: Imaging Neurosci (Camb). 2025 Jun 24;3:IMAG.a.52. doi: 10.1162/IMAG.a.52 (PMC12319828; doi:10.1162/IMAG.a.52)
Supplement: Supplementary Material [file imag.a.52_supp.pdf]

Supplements

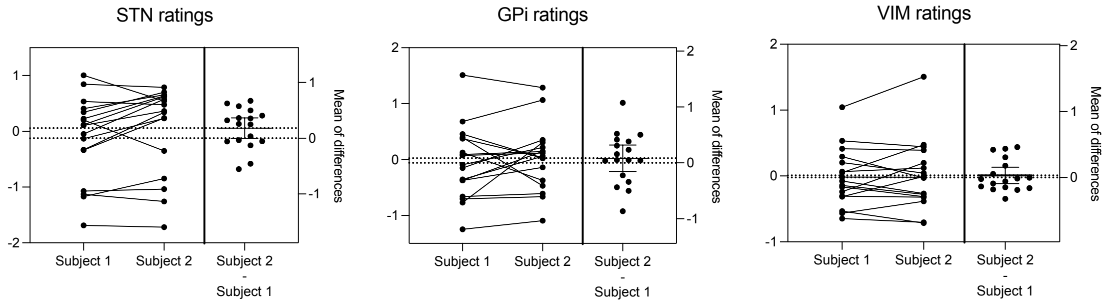

Fig. S1: Correlation and two-tailed paired t-test analyses of the ratings of all sequences scanned in both subjects showed a highly significant pairing between subjects (STN: R = 0.8924, p < 0.001; GPi: R = 0,7244, p < 0.0005, VIM: R = 0,8883, p < 0.0001). There was no significant difference between the two subjects (STN: p > 0.054; GPi: p > 0.47; VIM: p > 0.60).

Technique	ID	Overall rank	STN rank	GPi rank	VIM rank
**T2**	01	19	13	22	09
	02	09	07	13	08
	03	06	01	12	16
	04	04	02	04	06
	05	01	03	05	03
**T2FLAIR**	06	24	22	24	22
	07	18	17	14	18
	08	05	04	09	05
	09	14	11	15	11
**PD**	10	15	20	03	04
**T2***	11	12	12	11	13
	12	17	14	16	20
**SWI**	13	11	09	07	15
	14	16	15	17	12
	15	08	06	06	14
	16	13	10	10	17
	17	07	08	08	07
**T1 TIR**	18	23	24	21	19
**FGATIR**	19	02	21	02	02
	20	03	23	01	01
**mapping**	21	10 (QSM)	05 (QSM)	18 (QSM)	10 (QSM)
	21	20 (R2*)	16 (R2*)	20 (R2*)	21 (R2*)
	22	21 (QSM)	18 (QSM)	19 (QSM)	23 (QSM)
	22	23 (R2*)	19 (R2*)	23 (R2*)	24 (R2*)

Tab. S1: Rating based ranking (01: best, 24: worst) of individual sequences for targeting of the subthalamic nucleus (STN), globus pallidus internus (GPi), and ventrointermediate nucleus (VIM). Overall ranking is determined by averaging the mean rating results of all three target structures for any given sequence.

**ID**	**STN**			**GPi**			**VIM**		
	Mean	SD	SEM	Mean	SD	SEM	Mean	SD	SEM
01: T2	0,21	0,87	0,18	-0,73	0,63	0,13	0,10	0,72	0,15
02: T2	0,51	0,85	0,17	-0,07	0,98	0,20	0,11	0,83	0,17
03: T2	1,09	0,52	0,15	-0,04	0,88	0,25	-0,13	0,56	0,16
04: T2	0,78	0,53	0,11	0,24	0,75	0,15	0,29	1,46	0,30
05: T2	0,74	0,75	0,15	0,21	0,94	0,19	0,44	1,15	0,24
06: T2FLAIR	-1,10	0,68	0,14	-1,06	0,81	0,16	-0,66	0,56	0,12
07: T2FLAIR	-0,06	0,63	0,13	-0,09	0,76	0,16	-0,19	0,55	0,11
08: T2FLAIR	0,64	0,58	0,17	0,13	0,63	0,18	0,29	0,57	0,17
09: T2FLAIR	0,30	0,68	0,20	-0,14	0,63	0,18	0,05	0,61	0,18
10: PD	-0,99	0,49	0,10	0,83	1,08	0,22	0,33	1,35	0,27
11: T2*	0,28	0,81	0,17	0,04	0,74	0,15	0,01	0,74	0,15
12: T2*	0,13	0,62	0,13	-0,15	0,68	0,14	-0,25	0,65	0,13
13: SWI	0,38	0,65	0,13	0,15	0,67	0,14	-0,07	0,64	0,13
14: SWI	0,03	0,59	0,17	-0,15	0,68	0,20	0,04	0,76	0,22
15: SWI	0,52	0,57	0,17	0,16	0,70	0,20	-0,06	0,74	0,21
16: SWI	0,37	0,60	0,12	0,12	0,76	0,15	-0,18	0,82	0,17
17: SWI	0,51	0,71	0,15	0,14	0,86	0,18	0,13	0,84	0,17
18: TIR	-1,69	0,55	0,11	-0,62	0,67	0,14	-0,23	0,76	0,15
19: FGATIR	-1,05	0,68	0,14	1,33	1,07	0,22	1,07	1,19	0,24
20: FGATIR	-1,27	0,39	0,11	1,47	1,05	0,30	1,13	1,45	0,42
21: QSM	0,54	0,85	0,17	-0,17	0,81	0,16	0,10	1,13	0,23
21: R2*	0,00	0,78	0,16	-0,26	1,05	0,21	-0,52	0,63	0,13
22: QSM	-0,10	1,00	0,20	-0,19	0,97	0,20	-0,67	0,96	0,20
22: R2*	-0,23	0,74	0,21	-0,85	0,80	0,23	-0,94	0,50	0,15

Tab. S2: Mean, standard deviation and standard error of mean of all intra-rater z-scored sequence evaluations for targeting STN, GPi, and VIM.
